# Peste des Petits Ruminants Virus in Tibet, China

**DOI:** 10.3201/eid1502.080817

**Published:** 2009-02

**Authors:** Zhiliang Wang, Jingyue Bao, Xiaodong Wu, Yutian Liu, Lin Li, Chunju Liu, Longciren Suo, Zhonglun Xie, Wenji Zhao, Wei Zhang, Nan Yang, Jinming Li, Shushuang Wang, Junwei Wang

**Affiliations:** China Animal Health and Epidemiology Center, Qingdao, People’s Republic of China (Z. Wang, J. Bao, X. Wu, Y. Liu, L. Li, C. Liu, Z. Xie, W. Zhao, W. Zhang, N. Yang, J. Li, S. Wang, J. Wang); Tibet Center for Animal Disease Control, Lhasa, Tibet, People’s Republic of China (L. Suo); 1 These authors contributed equally to this article.

**Keywords:** Peste des petits ruminants virus, prevalence, ruminants, Tibet, China, dispatch

## Abstract

Serologic and molecular evidence indicates that peste des petits ruminants virus (PPRV) infection has emerged in goats and sheep in the Ngari region of southwestern Tibet, People’s Republic of China. Phylogenetic analysis confirms that the PPRV strain from Tibet is classified as lineage 4 and is closely related to viruses currently circulating in neighboring countries of southern Asia.

Peste des petits ruminants (PPR) is an acute and highly contagious viral disease contracted by small ruminants such as goats and sheep and causing high rates of illness and death. The disease is endemic in parts of sub-Saharan Africa, the Middle East, and Asia. The PPR virus (PPRV) genogroup consists of 4 lineages (*1*,*2*). PPRV infection was officially reported in the Ngari region of western Tibet, People’s Republic of China, in July 2007. Our study assesses the prevalence of PPRV infection in goats and sheep by region in Tibet. We also characterize strains of the virus by comparing part of the genome sequences with other PPRV sequences available in the GenBank database.

## The Study

Small ruminants in regions throughout Tibet were examined for PPRV antibody from July 2007 through November 2007. The sampling procedure focused on 3 groups of animals. The first comprised 718 animals in 4 counties (Rutog, Ge’gyai, Gerze, and Zada) in the Ngari region, where animals having clinical signs of PPRV infection had been reported by local authorities. The second group included 298 animals in Gar and Bulang counties in the same region and in 2 counties bordering the Ngari region (Nyima in Nagqu region and Zhongba in Shigatse region). The third group contained 520 animals in 5 counties within 3 separate regions (Nyalam and Yadong in Shigatse region, Cona and Lhozhag in Shannan region, and Zayu in Nyingchi region). We examined 1,536 animals (771 goats and 765 sheep) and collected serum samples from each. A competitive ELISA that used a monoclonal antibody to the N protein (*3*) identified 271 animals (17.6%) having antibody to PPRV. The PPRV-positive sera were collected from Rutog (122/209), Gerze (59/131), and Ge’gyai (90/314) counties in the Ngari region (Table 1). Rates of PPRV infection were higher in goats than in sheep. Of 763 goats examined in the Ngari region, 263 (34.5%) were seropositive for PPRV. The highest seroprevalence (61.6%, 121/198) was found in goats in Rutog County. Only 8 (11%) of 73 sheep examined in the Ngari region were seropositive for PPRV (Table 2).

**Table 1:** PPRV antibody in animals sampled in Tibet, China, 2007*

Region	County	No. samples	No. (%) PPRV positive
Ngari	Gerze	131	59 (45.0)
	Ge'gyai	314	90 (28.7)
	Rutog	209	122 (58.4)
	Zada	64	0
	Gar	50	0
	Bulang	68	0
Nyingchi	Zayu	60	0
Nagqu	Nyima	60	0
Shigatse	Nyalam	120	0
	Yadong	66	0
	Zhongba	120	0
Shannan	Cona	135	0
	Lhozhag	139	0
Total		1,536	271 (17.6)

*PPRV, peste des petits ruminants virus.

**Table 2:** Antibody response to PPRV by species in Tibet, China, 2007*

Species	Region	County	No. serum samples	No. (%) PPRV seropositive
Goat	Ngari	Rutog	198	121 (61.1)
		Gerze	126	56 (44.4)
		Ge'gyai	283	86 (30.4)
		Zada	61	0
		Gar	50	0
		Bulang	45	0
	Others		8	0
Sheep	Ngari	Rutog	11	1 (9.1)
		Gerze	5	3 (60.0)
		Ge'gyai	31	4 (12.9)
		Zada	3	0
		Gar	0	–
		Bulang	23	0
	Others		692	0
Total			1,536	271 (17.6)

*PPRV, peste des petits ruminants virus.

**Figure 1:**
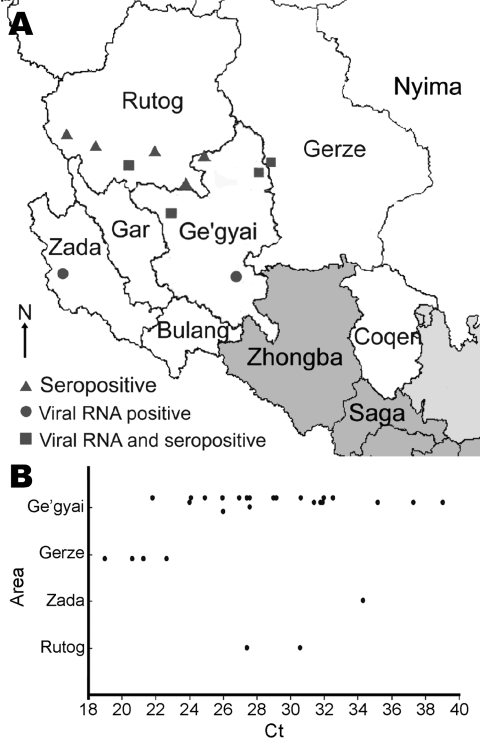
A) Distribution of outbreaks of peste des petits ruminants disease in Tibet, China, 2007. Triangles indicate outbreaks confirmed by ELISA. Circles indicate outbreaks confirmed by reverse transcription–PCR (RT-PCR) and quantitative RT-PCR. Squares indicate outbreaks confirmed by ELISA and molecular methods. B) Cycle threshold (Ct) values (determined by use of q-RT-PCRs on samples) by county.

Field samples, including organ (lymph node, spleen, lung, and intestine) and swab specimens, were obtained from 49 goats and sheep suspected of being infected with PPRV. These animals inhabited 4 counties in the Ngari region (Ge’gyai n = 33, Zada n = 7, Gerze n = 5, and Rutog n = 4). Two reverse transcription–PCRs (RT-PCR) and 1 newly developed and validated real-time quantitative RT-PCR (qRT-PCR) were conducted to determine whether the animals had viral RNA (*4*–*6*). The first RT-PCR (N RT-PCR), which amplified a 351-bp fragment in the N protein gene, detected virus in 28 samples. The second RT-PCR (F RT-PCR), which amplified a 448-bp fragment in the F protein gene, detected virus in 27 samples. The qRT-PCR detected virus in 37 samples. Use of qRT-PCR and 1 of the 2 RT-PCRs showed that 31 animals were found to contain viral RNA. In goats, 23 (77%) of the 30 samples contained viral RNA, and 2 (29%) of 7 sheep samples contained viral RNA. Most (61%) infected animals showed a high viral load with individual cycle threshold (Ct) values <30. Almost one third (29%) had a moderate viral load (Ct 30–35), and 10% had a Ct value >35. The distribution of Ct values differed slightly according to the infected animal’s origin. All animals from Gerze County had low Ct values (Ct 19–23), indicating high viral loads. However, no animal from Zada County had a Ct <30 (Figure 1).

The study confirmed 11 outbreaks in 4 counties in the southwest Ngari region in Tibet. Nine of the 11 occurred in southern Rutog County, northern Ge’gyai County, and Gerze County, and samples from these 9 were seropositive for PPRV (Figure 1, panel A). By using qRT-PCR and RT-PCR, we found that samples from 4 of these outbreaks were also seropositive for PPRV virus RNA. This finding, confirmed by sequencing, indicates that the main portal of entry for the disease is through southwestern Rutog County. Viral RNA was also detected and confirmed by sequencing in 1 outbreak in southern Ge'gyai County and another outbreak in Zada County (Figure 1).

**Figure 2:**
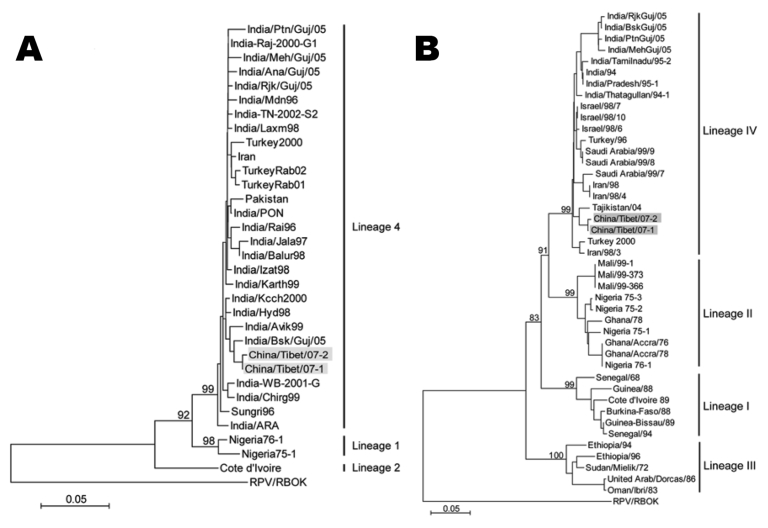
Phylogenetic relationship between peste des petits ruminants virus (PPRV) detected in Tibet, China, in 2007 and other virus isolates. PPRV strains sequenced in this study are highlighted in gray. Other sequences are from GenBank. Phylogenetic analyses were completed with MEGA 3.1 software that used a neighbor-joining algorithm and absolute distances and that followed 1,000 bootstrap replicates. The RBOK vaccine strain of rinderpest virus was included as an outgroup. The tree is based on the partial sequence of the fusion (F) protein gene (A) and the nucleocapsid (N) protein gene (B). Different classifications were used for the phylogenetic comparisons for the West African lineages 1 and 2. Nigeria and related strains have been classified as lineage 1; the Côte d’Ivoire and related strains have been classified as lineage 2 (*1*). Later research reversed this order in classifying the lineages in N gene analyses (*2*).

The nucleic acid sequences obtained from the PCR products were aligned with sequences from PPRV strains available in GenBank. Partial sequencing (448 bp) of the F gene showed that 20 of 21 samples were identical over the portion of the genome that was characterized (GenBank accession no. EU816772). One (GenBank accession no. EU815053) differed from other Ngari sequences by 1 nt. The Ngari sequences showed a level of nucleotide identity with other PPRV strains of 88.8%–98.8%. Strains of PPRV from Tibet were classified as lineage 4 and were closely related to the India/Bsk/Guj/05 strain isolated in India in 2005 (Figure 2, panel A). Partial N-gene sequences (351 bp) from 18 of 19 Ngari samples were identical (GenBank accession no. EU068731), and 1 sequence (GenBank accession no. EU340363) differed by 1 nt. Sequence comparison of the Ngari N gene to the sequences of other PPRV strains showed a nucleotide identity level of 81.6%–97.3%. Strains of PPRV from Tibet were classified as lineage IV and were closely related to the Tajikistan/04 isolate found in Tajikistan in 2004. Different kinds of numbering were used for the phylogenetic comparisons for the 4 lineages of these 2 genes. The lineages were classified as 1–4 in F gene analyses (*1*). Later research classified the lineages in N gene analyses as I–IV (*2*) (Figure 2, panel B).

## Conclusions

In this study, PPRV was found by collecting samples from animals in the field and detecting infection by using competitive ELISA and RT-PCR. Our research provides valuable data on PPRV infection in small ruminants in Tibet. Infection was observed in 4 counties in the Ngari region of southwestern Tibet. Most outbreaks occurred in Rutog and Ge’gyai counties; 1 outbreak was confirmed in Gerze County and another in Zada County. Epidemiologic and serologic evidence suggests that the infection first emerged in Rejiao village in southwestern Rutog from November 2005 through March 2006. PPR likely existed for several years without being recognized in Tibet because veterinarians, animal health workers, and livestock owners in the area are unfamiliar with its clinical and pathologic features. Also, this disease is frequently confused with other diseases that cause respiratory problems and death in small ruminants (*7*).

The molecular epidemiologic techniques provided data suggesting cross-border transmission of PPRV infection into Tibet. PPR infection has been recognized in many Asian countries bordering southwestern China, including India (*8*), Nepal (*9*), Bangladesh (*9*), Pakistan (*10*), and Afghanistan (*7*). Almost all recent viruses from southwest Asia and the Middle East belong to PPRV lineage 4. The virus that circulated in the Ngari region is of the same lineage and is closely related to an isolate from India (2005) and an isolate from Tajikistan (2004). Close contact between susceptible animals and infected animals in the febrile stage is the main method of transmitting PPR. The terrain of western and southwestern Ngari permits uncontrolled animal movement, and a small ruminant trade exists between Tibet and bordering nations such as India and Nepal. These factors and the history of PPRV in Asia suggest that animals from a neighboring country in southwest Asia are likely sources of this infection in Tibet.
